# Beauveria attenuates asthma by inhibiting inflammatory response and inducing lymphocytic cell apoptosis

**DOI:** 10.18632/oncotarget.12958

**Published:** 2016-10-27

**Authors:** Jingying Zhang, Xianmei Zhou, Jiping Zhu

**Affiliations:** ^1^ Department of Respiratory Medicine, Affiliated Hospital of Nanjing University of Traditional Chinese Medicine, Nanjing, China

**Keywords:** asthma, beauveria, inflammation, lymphocytes, cell apoptosis, Pathology Section

## Abstract

The present study aimed to investigate the role of beauveria (BEA) in asthma. We investigated the cytotoxic effect of BEA on the proliferation of inflammatory cells and secretion of inflammatory mediators both in-vitro and in-vivo. In in-vitro studies, BEA inhibited lymphocytic cell proliferation and the proliferation of lymphocytic cells induced by Phorbol-12-myristate-13-acetate (PMA). We used ELISA to test the effects of BEA on the secretion of inflammatory factors including tumor necrosis factor-alpha (TNF-α), interleukin-12 (IL-12) and interferon-gamma (IFN-γ). Flow cytometry was used to evaluate the influence of BEA on cell apoptosis. The effect of BEA on the cell numbers of eosinophils, lymphocytes, macrophages, neutrophils and other cells in mouse bronchoalveolar lavage fluid (BALF) was also evaluated. The expression of apoptosis related molecules Bax, Caspase-3 and Bcl-2 was examined by Western blotting analysis. Our results indicated that BEA played a protective role in asthma. BEA inhibited lymphocytic cell proliferation and secretion of inflammatory mediators. BEA promoted cell apoptosis, stimulated the expression of Bax and Caspase-3 and inhibited Bcl-2 protein expression in a dose-dependent manner. In in-vivo experiments, BEA reduced the cell number of eosinophils, lymphocytes, macrophages, neutrophils and other cells in mouse BALF. BEA inhibited secretion of inflammatory mediators, stimulated expression of Bax and Caspase-3, and inhibited expression of Bcl-2 in mouse lung tissue dose-dependently. All together, our results indicated that BEA could attenuate asthma by inhibiting inflammatory response and induce apoptosis of inflammatory cells.

## INTRODUCTION

Asthma is a common chronic inflammatory disease in the respiratory tract characterized by variable/recurring symptoms of reversible airflow obstruction and bronchospasm [1]. It is caused by a combination of genetic and environmental factors [2]. The inflammaroty cells such as lymphocytes, macrophages and neutrophils are involved in the pathogenesis of asthma. Components of the immune system mediators including cytokines, chemokines, histamine and leukotrienes are contributing factors of asthma [3]. The incidence of asthma has increased greatly since the 1970s. In 2011, it was diagnosed in 235-300 million people [4, 5] and caused 250,000 deaths globally [5].

Despite the various treatment options currently available for the therapeutic management of asthma, a large number of patients with asthma continue to have poorly controlled disease. There is therefore a need of novel medication to achieve better asthma control.

Beauveria (BEA) is a genus of asexually-reproducing fungi belongs to the ascomycete family cordycipitaceae [6]. The role of BEA in inhibiting inflammation was previously reported in CD-1 mice [7, 8]. Many plant species containing flavonoids has been widely used as traditional Chinese medicine. Naringin, which possesses antioxidant, anti-inflammatory and anti-carcinogenic properties, was previously reported to protect ovalbumin-induced airway inflammation in asthma [9]. Moreover, naringin was found to attenuate ovalbumin induced cough-variant asthma in a guinea pig model [10]. As inflammation and its relationship to asthma are becoming better understood, the importance of anti-inflammatory treatment is increasingly accepted.

However, whether BEA could be a medication for asthma is not clear. The present study focuses to investigate the effect of BEA on the growth of inflammatory cells and secretion of inflammatory mediators. The aim is to investigate if BEA plays a protective role in asthma.

## RESULTS

### BEA inhibits the growth of lymphocytes in a dose-dependent manner

**Figure 1:**
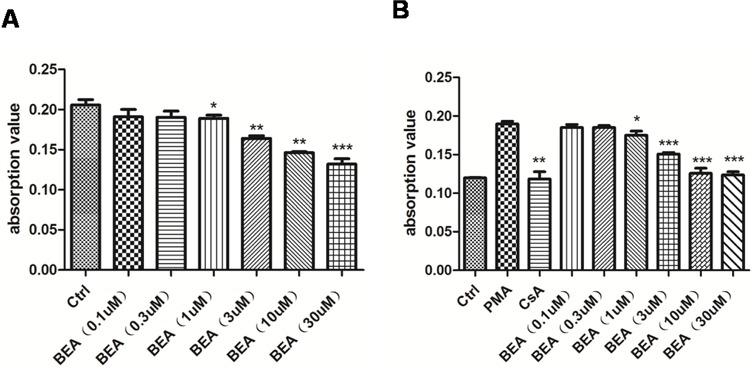
BEA inhibited PMA-induced lymphocytes proliferation in a dose-dependent manner **A.** The inhibition effect of BEA on the proliferation of lymphocytes. Data are presented as mean±SD of triplicates. Statistical analysis between control group (Ctrl) and BEA treatment groups at indicated concentrations was determined by student's t-test. Significance level was labeled as: * *P* <0.05; ** *P* <0.01; *** *P*<0.001. **B.** The inhibition effect of BEA on the PMA-induced lymphocytes proliferation. Data are presented as mean±SD of triplicates. Statistical analysis between PMA group and CsA or BEA treatment groups was determined by student's t-test. Significance level was labeled as: * *P* <0.05; ** *P* <0.01; *** *P*<0.001.

The cytotoxicity of BEA was investigated by treating lymphocytic cells isolated from mesenteric lymph nodes with different concentration of BEA. It was found that low doses of BEA (less than 0.3 μM) did not affect lymphocytic cell growth, while high dose of BEA (greater than 1 μM) significantly inhibited lymphocytic cell proliferation. The higher the BEA concentration, the lower the cell proliferation as reflected as lower light absorbance value (Figure 1A). This result indicated that BEA inhibited the growth of lymphocytes in a dose-dependent manner.

### BEA inhibits PMA-induced lymphocytes proliferation dose-dependently

It was reported that the proliferation of lymphocytes could be induced by PMA [11], and PMA-induced lymphocytic proliferation could be inhibited by Cyclosporine A (CsA) [12]. In our experiments, the proliferation of lymphocytes increased greatly when cells were treated with PMA, and the PMA-induced lymphocytic proliferation was inhibited by CsA treatment (Figure 1B). We found that low doses of BEA (less than 0.3 μM) did not affect the PMA-induced lymphocytic proliferation, while high dose of BEA (greater than 1μM) significantly inhibited PMA-induced cell proliferation. The higher the BEA concentration, the higher inhibition of cell proliferation (Figure 1B). This result indicated that BEA inhibited the PMA-induced lymphocytic cell proliferation in a dose-dependent manner.

### BEA inhibits PMA induced up-regulation of inflammatory mediators dose-dependently

Since BEA inhibited the growth of lymphocytes and PMA-induced lymphocytes proliferation, we examined if BEA could inhibit the release of inflammatory mediators secreted by immune cells. TNF-α, IL-12, and IFN-γ were chosen because they are important mediators of inflammatory responses and represent the function of different immune cells. TNF-α is produced mainly by macrophages; IL-12 is produced by dendritic cells and macrophages, and increases the number of Th2 cells; and IFN-γ is the principle Th1 effector cytokine. BEA's effect on inflammatory response is not by inhibiting a single type of cell or cytokine, but by regulating proliferation and function of multiple cells. Compared with the control group, the secretion of inflammatory factors such as TNF-α, IL-12 and IFN-γ were significantly up-regulated in PMA treated cells, and decreased in CsA treated cells. The results showed that high dose of BEA (>3 μM) notably decreased PMA induced up-regulation of TNF-α, IL-12 and IFN-γ. The inhibitory effect of BEA was not notable in cells treated with low dose (1μM) of BEA (Figure 3). This result indicated that BEA inhibited the PMA-induced up-regulation of inflammatory factors released by immune cells dose-dependently.

**Figure 2:**
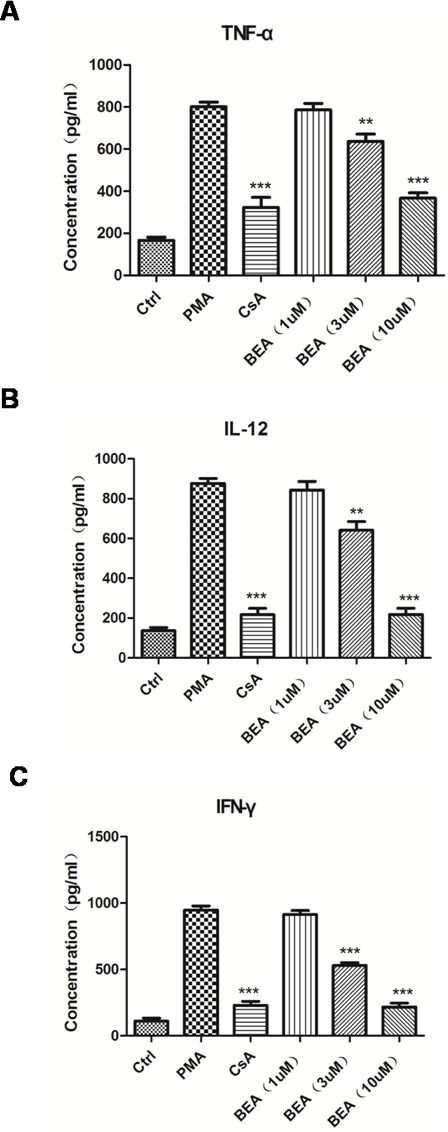
BEA inhibited PMA-induced up-regulation of inflammatory mediators of lymphocytes The levels of TNF-α **A.**, IL-12 **B.** and IFN-γ **C.** released by lymphocytes under different treatments were examined using ELISA kits as described above. Data are presented as mean±SD of triplicates. Statistical analysis between PMA group and CsA or BEA treatment groups was determined by student's t-test. Significance level was labeled as following: ** *P* <0.01; *** *P* <0.001.

**Figure 3:**
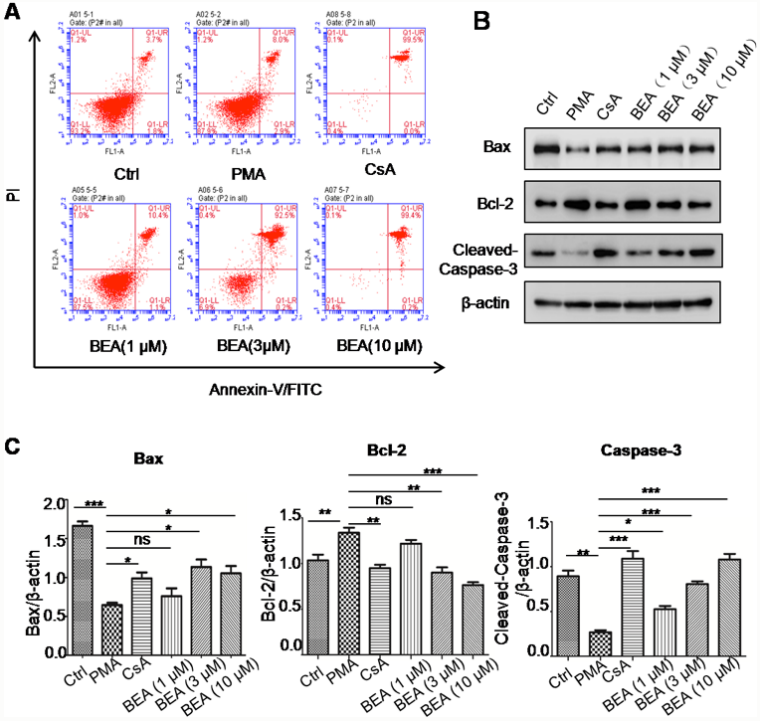
BEA inhibited PMA-induced cell apoptosis by up-regulating Bax and caspase-3 and down-regulating Bcl-2 in lymphocytes **A.** Lymphocytes under different treatments were stained with Annexin-V and PI and analyzed by Flow cytometry. **B.** Western blotting analysis of Bax, Bcl-2 and cleaved Caspase-3 in lymphocytes. **C.** Quantitative analysis of Western blotting results. Specific protein expressions were normalized to the levels of β-actin. Data are presented as mean±SD of three experiments. Statistical analysis between highlighted groups was determined by student's *t*-test. Significance level was labeled as: ns; *P* >0.05; * *P* <0.05; ** *P* <0.01; *** *P* <0.001.

### BEA induced cell apoptosis dose-dependently by stimulating expression of Bax and Caspase-3, and inhibiting expression of Bcl-2 in lymphocytes

The effect of BEA on lymphocytic cell apoptosis was investigated. Although BEA at 1μM did not alter the status of cell apoptosis, BEA at 3, 10 and 30μM significantly induced cell apoptosis dose-dependently (Figure 3A). Western blotting analysis showed that in PMA treated lymphocytic cells, protein levels of Bax and Cleaved-Caspase-3 were significantly lower than those in control cells, and Bcl-2 protein level was significantly higher than in control cells. CsA reversed the effects of PMA. Furthermore, BEA (1, 3 and 10μM) dose-dependently promoted expression of Bax and Caspase-3, and inhibited expression of Bcl-2 (Figure 3B). The quantitative analysis of protein expression was shown in Figure 3C.

### BEA reduced the cell number of eosinophil, lymphocyte, macrophage, neutrophil and total cells in mice bronchoalveolar lavage fluid (BALF)

**Figure 4:**
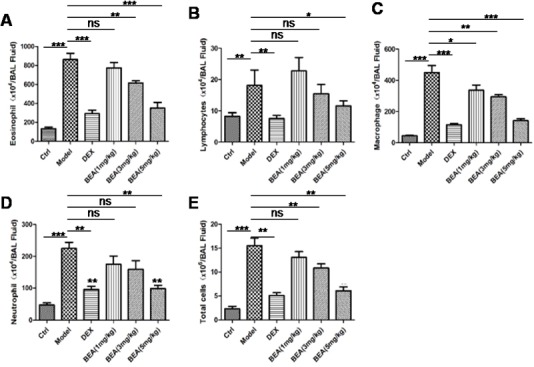
BEA reduced the numbers of eosinophils, lymphocytes, macrophages, neutrophils and total cells in mice BALF The numbers of eosinophils **A.**, lymphocytes **B.**, macrophages **C.**, neutrophils **D.** and total cells **E.** in mice BALF. Data are presented as mean±SD of 8 mice each group. Statistical analysis between highlighted groups was determined by student's *t*-test. Significance level was labeled as: ns; *P* <0.05; * *P* <0.05; ** *P* <0.01; *** *P* <0.001.

PMA treatment notably induced the cell number of eosinophil, lymphocyte, macrophage, neutrophil in mice BALF. The number of all cells (Total cells) including both inflammatory cells and other cells in BALF were increased. Dexamethaone (DEX) treatment decreased both inflammatory cells as well as other cells. BEA treatment showed similar inhibitory effect as DEX, showing that BEA at 1 mg/kg slightly decreased cell number, while at the concentration of 3 and 5 mg/kg significantly deceased the cell number in BALF (Figure 4).

### BEA inhibited PMA induced up-regulation of inflammatory mediators dose-dependently in asthma mouse model

**Figure 5:**
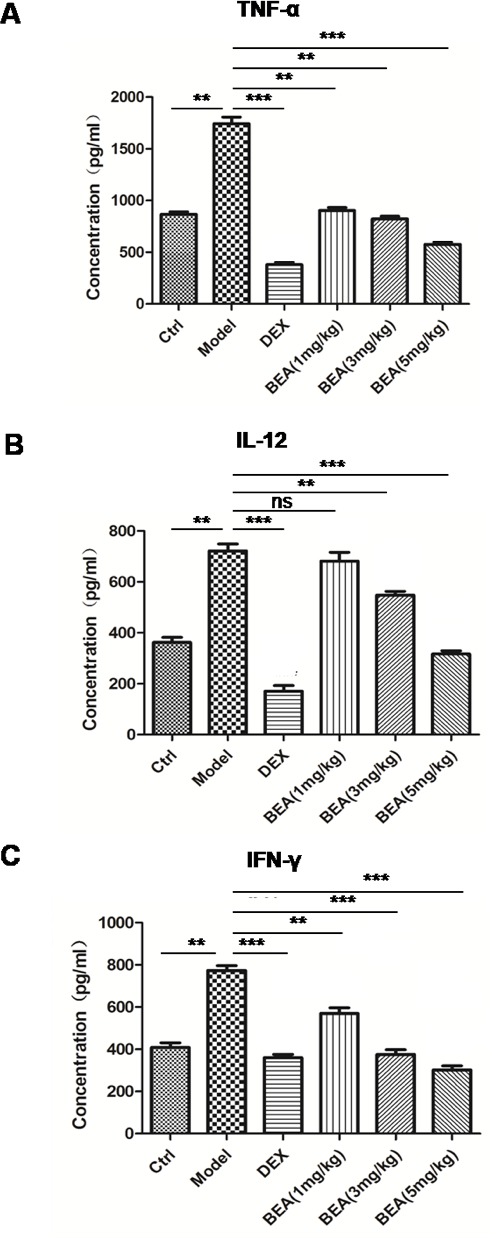
BEA reduced serum levels of TNF-α, IL-12 and IFN-γ in mice The levels of TNF-α A., IL-12 **B.** and IFN-γ **C.** were assessed by ELISA kits as described. Data are presented as mean±SD of 8 mice each group. Statistical analysis between highlighted groups was determined by student's *t*-test. Significance level was labeled as: ns; *P* >0.05; ** *P* <0.01; *** *P* <0.001.

The influence of BEA on the serum levels of inflammatory mediators was investigated in an asthmas mouse model. Compared with control group, levels of TNF-α and IFN-γ in serum were significantly up-regulated in PMA treated mice, and DEX decreased the levels PMA up-regulated inflammatory factor significantly. High dose of BEA (5 mg/kg), but not low dose of BEA (1 and 3 mg/kg), notably decreased PMA induced up-regulation of TNF-α. Similarly, PMA induced up-regulation of IFN-γ was notably decreased by BEA (1, 3 and 5mg/kg) treatment in a dose-dependent manner (Figure 5).

### BEA increased expression of Bax and Caspase-3 and decreased expression of Bcl-2 in lung tissue

**Figure 6:**
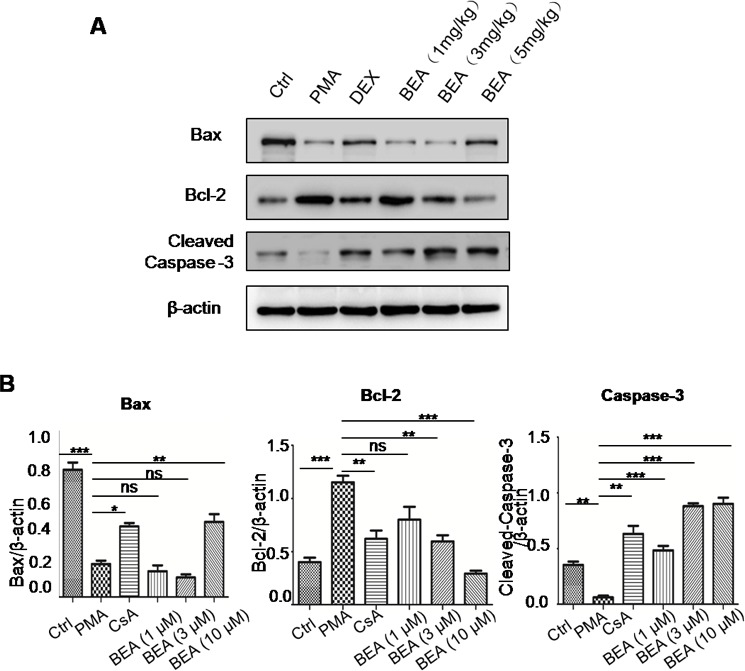
Western blotting analysis of Bax, Caspase-3 and Bcl-2 in mouse lung tissues **A.** The western blotting results of representative animals from different treatment groups. **B.** Quantitative analysis of Western blotting results. Specific protein expression levels were normalized to the levels of β-actin. Data are presented as mean±SD of three experiments. Statistical analysis between highlighted groups was determined by student's *t*-test. Significance level was labeled as following: ns; *P* >0.05; * *P* <0.05; ** *P* <0.01; *** *P* <0.001.

The expression of Bax, Caspase-3 and Bcl-2 of lung tissue of asthma mice was analyzed using Western blotting. The results showed that in the lung tissue of PMA treated mice, protein levels of Bax and Caspase-3 were lower than those of control group and Bcl-2 protein level was higher than control group. DEX reversed the effects of PMA. BEA (1, 3 and 5mg/kg) dose-dependently promoted the expression of Bax and Caspase-3 and inhibited the expression of Bcl-2 in lung tissue (Figure 6A). The quantitative analysis of protein expression was shown in Figure 6B.

### BEA decreased pulmonary inflammatory cell infiltration and collagen deposition

**Figure 7:**
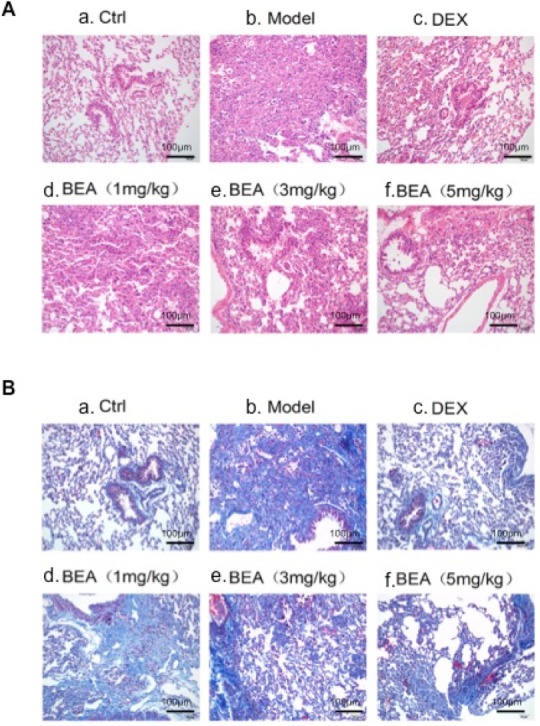
H&E and Masson Trichrome staining of mouse lung tissue **A.** HE staining. a. Normal lung with no inflammatory cells. b. Asthmas lung with a number of inflammatory cell infiltration and collagen deposition. c. DEX treated lung with a few inflammatory cell deposition and collagen deposition. d. 1mg/kg BEA treated lung with a number of inflammatory cell infiltration and collagen deposition. e. 3mg/kg BEA treated lung with some inflammatory cell infiltration and collagen deposition. f. 5mg/kg BEA treated lung with few inflammatory cell infiltration and few collagen deposition. **B.** Masson Trichrome staining. a. Normal lung with few collagen around the bronchial and blood vessels. b. Asthmas lung with a number of inflammatory cell infiltration and dense collagen deposition. c. DEX treated lung with some collagen deposition. d. 1mg/kg BEA treated lung with collagen deposition. e. 3mg/kg BEA treated lung with a few collagen deposition. f. 5mg/kg BEA treated lung with few collagen deposition.

Normal lung tissue had few inflammatory cell infiltration and collagen was seem only around the basement membrane of bronchial epithelia and blood vessels. In mice with asthmas, lung tissue had increased inflammatory cell infiltration and deposition of collagen were seen not only in basement membrane of bronchial epithelia and blood vessels, but also in lung parenchyma, indicating the increased proliferation of fibroblasts and increased secretion of collagen. DEX treatment decreased inflammatory cell infiltration and collagen deposition. Low dose BEA (1mg/kg) had little effect in reducing inflammatory cell infiltration and collagen deposition. However, high dose BEA (3mg/kg) reduced the inflammatory cell infiltration and collagen deposition. BEA at 5mg/kg greatly reduced the inflammatory cell infiltration and collagen deposition (Figure 7).

## DISCUSSION

Asthma is a common chronic inflammatory disease in the airways [2]. Precipitous symptomatic attacks of asthma may be caused by exposure to factors such as: allergens [13], viruses [14] and indoor/outdoor pollutants [15]. These factors often induce an acute inflammatory response. Eosinophils, lymphocytes, macrophages, neutrophils, as well as inflammatory mediators including cytokines, chemokines, histamine and leukotrienes participate in the process of asthma [2].

BEA is a genus of asexually-reproducing fungi allied with the ascomycete family cordycipitaceae [6]. The role of BEA in inhibiting inflammation was previously reported in CD-1 mice [7, 8]. As the relationship between inflammation and asthma are becoming better understood, the importance of anti-inflammatory treatment is increasingly accepted. Whether BEA plays a protective role in asthma via inhibiting inflammatory response is unknown.

In the present study, we found that BEA inhibited the proliferation of inflammatory cells as well as the secretion of inflammatory mediators. It also inhibited the proliferation of inflammatory cells and secretion of inflammatory factors induced by PMA. These results suggested that BEA played a protective role in PMA-induced asthma by inhibiting inflammation. These results were consistent with previous studies that showed an association between the extent of inflammation and the clinical severity of asthma [16, 17].

The influence of BEA on immune cells was further tested. In-vitro study showed that PMA significantly increased cell proliferation of lymphocyte. We found that although low doses of BEA (0.1 and 0.3 μM) did not affect cell proliferation, high doses of BEA (1, 3, 10 and 30 μM) dose-dependently decreased cell proliferation of lymphocyte induced by PMA. In-vivo study showed that PMA notably induced the cell number of eosinophil, lymphocyte, macrophage, neutrophil and other cells in mice BALF, this up-regulation of inflammatory cells was inhibited by DEX as well as by BEA (1, 3 and 5 mg/kg) dose-dependently. These results were consistent with previous studies that showed the severity of asthma was correlated with inflammatory indices such as increased the number and activation of eosinophils [16, 18], T-cells [19]and macrophages [19, 20].

Apoptosis, a dynamic process involved in the control of the “tissue load” of cells at inflamed sites, tends to limit inflammatory tissue injury and promote resolution but not inflammation progression [21, 22]. We found that BEA at concentration of 1, 3, 10 and 30μM significantly induced cell apoptosis in a dose-dependent manner.

We investigated the expression of apoptotic-related molecules by Western Blotting analysis. We found that in in-vitro cultured lymphocytes, protein levels of Bax and Cleaved-Caspase-3 were significantly lower in PMA group than that in control group; while Bcl-2 protein level was significantly higher than control group. BEA (1, 3 and 10μM) dose-dependently stimulated the expression of Bax and Cleaved-Caspase-3 and inhibited the expression of Bcl-2. Similarly, in the lung tissues of asthma mice, the protein levels of Bax and Cleaved-Caspase-3 were significantly lower in the PMA group than those of control group; and Bcl-2 protein level was significantly higher than control group. BEA (1, 3 and 5mg/kg) dose-dependently stimulated the expression levels of Bax and Cleaved-Caspase-3, and inhibited expression of Bcl-2 in lung tissue.

Our study indicated that BEA could potentially played an important role in inhibiting inflammation and preventing the development of asthma. This hypothesis is based on the following findings: in in-vitro experiments, BEA inhibits PMA induced up-regulation of inflammatory cells such as lymphocytes as well as inflammatory mediators. BEA induces cell apoptosis in a dose-dependent manner by promoting the expression of Bax and Cleaved-Caspase-3, and decreasing Bcl-2 protein expression. In in-vivo experiments, BEA reduces cell number of inflammatory cells such as eosinophil, lymphocyte, macrophage, neutrophil and other cells in mice BALF. Similarly, BEA inhibits PMA induced up-regulation of inflammatory mediators, increases expression of Bax and Caspase-3 in a dose-dependent manner, inhibits PMA induced up-regulation of expression of Bcl-2 in lung tissues. Taken together, BEA could attenuate asthma through inhibiting inflammatory response and promoting apoptosis of inflammatory cells.

In conclusion, our results suggested that BEA played a protective role in asthma by inhibiting inflammation through decreasing proliferation and increasing apoptosis of inflammatory cells. We demonstrated that the function of BEA in inhibiting inflammation response is by targeting apoptosis-related molecules. These results provided a rationale for the application of BEA in the treatment of asthma.

## MATERIALS AND METHODS

### Chemicals and reagents

BEA (Beauvericin), MSS1033-1 was purchased from Pte. Ltd. (QingDao, China), RPMI-1640 from Hyclone (Logan, UT), Bovine fetal serum from BI (Israel). Mouse IL-12, IFN-γ and TNF-α ELISA kits were from Elabscience Biotechnology Co. Ltd, (Wuhan, China) and FITC-anti-mouse was from KeyGEN BioTECH (Nanjing, China)

### Isolation of lymphocytes

Mesenteric lymph nodes of adult BALB/c mice were isolated under sterilized condition, placed in RPMI-1640 culture medium (Hyclone, Logan, UT), minced gently and beat up with pipette tips to disperse cells. Then cells were filtered with 37 μm diameter filter screen (BD, NJ) followed by centrifugation at the speed of 1000rpm for 5min at 4°C. Finally, the supernatant was discarded and cells were suspended in RPMI-1640 culture medium and cultured at a 37°C incubator which was supplemented with 5% CO_2._

### Establishment of asthmas mouse model

This study was approved by the Ethics Committee of Jiangsu Drug Research Institute. All animal experiments followed the guideline of On the Care and Use of Laboratory Animals of the Institute. Adult female BALB/c mice 6-8 weeks old weighing 18-22g were used in this study. All mice were housed in a pathogen-free conditions with controlled temperature (21 ± 2°C) and 12h light-dark cycle. Mice were matched based on age and body weight. They were randomly divided into 6 groups with 8 mice in each group: Group I- control group; Group II-asthma mice; Group III- asthma + DEX (10 mg/kg); Group IV-asthma +BEA (1mg/kg); Group V-asthma +BEA (3mg/kg); and Group VI-asthma +BEA (5mg/kg). Asthmas mouse models were established by intraperitoneal injection of 0.5 ml sensitization liquid on the 1^st^ day, and the injection was repeated on the 8^th^day. Sensitization liquid consisted of 0.8 mg OVA (ovalbumin Grave V, Sigma) and 40 mg Al(OH)_3_ in 20ml saline. Mice in control group were injected with 0.5 ml saline. Asthmas motivate solution consisted of 1g OVA (Grave II, Sigma) in 100ml saline. Mice were motivated every day by automatic inhalation of motivate solution for 30min from the 21^st^ to 25^th^ day. In the control group, mice inhaled saline instead of motivate solution. In the treatment groups, DEX (10 mg/kg) and 3 different dosages of BEA (1, 3 and 5mg/kg) were given by intraperitoneal injection 30min before automatic inhalation of motivate solution.

### Cell proliferation assay

The effect of BEA cytotoxicity was evaluated by determining the cell proliferation capacity using CCK-8 assay. Lymphocytic cells isolated from mesenteric lymph nodes were seeded into 96-well plate at the concentration of 5×l0^5^cells/well. They were treated with different concentrations of BEA (0.1, 0.3, 1, 3, 10 and 30 μM) and cultured in 37°C incubator supplemented with 5% CO2 for 24 h. Afterwards, 10 μl CCK-8 (Vazyme biotech Co., Ltd, Nanjing, China) was added and cultured for another 1h. OD value of the culture medium was read at 450nm using a Bio-Rad iMark plate reader. The cytotoxicity of BEA was evaluated based on the OD values.

The effects of PMA (Phorbol-12-myristate-13-acetate) on the proliferation of lymphocytes were also tested by CCK-8 assay. Lymphocytic cells were first cultured in the medium supplemented with 50ng/ml PMA for 6h, then seeded into 96-well plate at the concentration of 5×l0^5^ cells/well and culture in 37°C incubator which was supplemented with 5% CO_2_for 72h. Afterwards, 10 μl CCK-8 was added and cultured further for another 1h. OD value was read and evaluated as above.

### Cell apoptosis assay

Cell apoptosis was evaluated by flow cytometric assay. 2×10^6^ cells in each group were used. Cells were centrifuged at 1000rpm for 5 min at 4°C and supernatant was discarded. Cells were then washed with pre-cold PBS twice, placed in 100 μl Binding Buffer, added with 5 μl Annexin-V-HTC (KeyGEN BioTECH, Nanjing, China)and mixed. Afterwards, the mixture was placed on ice for 15min and 400 ul PBS was added into the mixture and proceed for Epics XL flow cytometric analysis (Beckman Coulter,CA,USA).

### ELISA

The protein levels of TNF-a, IL-12 and IFN-γ in the supernatant of cell culture or mouse serum samples were assessed by Mouse ELISA kits according to manufacture's instructions. Briefly, 96-well plates were coated with TNF-a, IL-12 or IFN-γ coating antibodies and placed overnight at 4°C. After that, plates were blocked by 2% BSA for 2 h at 37°C. Different cell supernatants or mouse serum samples (diluted 1:10 in assay diluent) were added to wells and incubated for 1 h at room temperature. Recombinant cytokine was assayed for generating standard curve and calibrating samples. After wash, corresponding biotin-conjugated antibodies, diluted at 1:5000, were added to the plate for 1 h at 37°C. Then, the plates were incubated with streptavidin-peroxidase 1:1000 for 30 minutes at 37°C. After 3 times washes, the substrate 3,39,5,59-tetramethylbenzidine (TMB) in citrate buffer was added to the corresponding plates. Reactions were stopped by 2N H2SO4. Optical densities were read by a ELISA reader (Thermo fisher, China) at 450 nm. Each sample was assayed in triplicate.

### Western blotting analysis

To identify the expression profiles of apoptosis related proteins Bax, Bcl-2 and caspase3, lysates of cultured lymphocytes and lung tissues were collected and analyzed. Briefly, samples were washed with PBS and homogenized in RIPA lysis buffer containing a cocktail of protease inhibitors and phosphatase inhibitors [2, 3]. Sample lysates were separated by SDS-PAGE and electronically transferred onto polyvinylidene fluoride membranes. Membranes were blocked in 5% BSA for 1 h at room temperature, incubated overnight at 4°C with the corresponding primary antibodies respectively. After washing with TBST, membranes were incubated with corresponding HRP-conjugated secondary antibodies for 1 h at room temperature. Detection of β-actin was used as a loading control.

### Hematoxylin and eosin (HE) staining

Lung tissues were fixed with 4% paraformalde­hyde, embedded in paraffin, sliced into 5 μM sections and stained with HE. Briefly, slices were dewaxed in xylene and rehydrated by graded alcohols. After washing with running water and distilled water, they were stained with hematoxylin for 3-5 min. Slides were washed in PBS again and clear in 1% HCl in 70% alcohol. Slices were further stained with eosin for 1-4 min. After dehydration in graded concentration of alcohols, slices were cleared in xylene and mounted in paramount. Slides were studied under a light microscope.

### Masson trichrome staining

Masson Trichrome staining was performed using a staining kit from Shanghai Bogoo Biotechnology.Co,Ltd, (Shanghai, China) according to the manufacturer's instruction. Briefly, tissues were fixed in Bouin solution and proceeded for routine paraffin embedding, sectioning and trichrome staining.

### Statistical analysis

All results are presented as means ± SD. Comparisons between groups were analyzed by two-way analysis of variance (ANOVA). *P* < 0.05 indicated a statistical difference.
